# Differential associations between synthetic endocrine disruptors and renal function impairment across varying degrees in middle-aged and older adult populations

**DOI:** 10.3389/fpubh.2025.1526070

**Published:** 2025-07-10

**Authors:** Junfang Mu, Yajing Li, Lu He, Mingyan Ma, Mohan Zhang, Xin Wang, Danhui Mao

**Affiliations:** ^1^Management School, Shanxi Medical University, Taiyuan, China; ^2^Department of Nephrology, Shanxi Bethune Hospital, Shanxi Academy of Medical Sciences, Tongji Shanxi Hospital, Third Hospital of Shanxi Medical University, Taiyuan, China; ^3^Department of Urology, First Hospital of Shanxi Medical University, Taiyuan, China; ^4^Department of Computer Science and Technology, Taiyuan University of Technology, Taiyuan, China

**Keywords:** synthetic endocrine disruptors, renal function, perfluoro/polyfluoroalkyl compounds, phosphorus-containing pesticides, plasticizers

## Abstract

**Background:**

Existing research has revealed that various factors, including environmental pollutants, can affect renal function. This study aims to explore the association between various synthetic Endocrine Disruptors(EDs), which are widely present in the environment, and the renal function of middle-aged and older adult populations.

**Methods:**

Based on data from the National Health and Nutrition Examination Survey (NHANES) from 2015 to 2018, a total of 1,199 participants, with a median age of 57 years, who had CKD or at least one CKD complication (such as dyslipidemia, hypertension, diabetes, or hyperuricemia), were included. A comprehensive assessment was conducted on the relationship between 28 synthetic EDs (including perfluoro/polyfluoroalkyl compounds, phosphorus-containing pesticides, and plasticizers) and renal function. Serum concentrations of synthetic EDs were determined using mass spectrometry. Renal function was assessed through urine Albumin-to-Creatinine Ratio (ACR) measurements. The quantile regression models were employed to explore the association between synthetic EDs and renal function. The grouped regression models were used to investigate the differential associations of synthetic EDs with non-CKD and CKD populations.

**Results:**

In this study, N-perfluorooctanoic acid, MECP phthalate, and Diethylthiophosphate were found to exert damaging effects on renal function. N-perfluorooctanoic acid and MECP phthalate were observed to exhibit differential associations with non-CKD and CKD populations. There was a significant difference in the effect of N-perfluorooctanoic acid between the non-CKD and CKD groups. N-perfluorooctanoic acid was not associated with renal function impairment in the non-CKD group, whereas it exhibited a significant adverse association in the CKD group. There was a significant difference in the effect of MECP phthalate between the non-CKD and CKD groups. MECP phthalate was associated with renal function impairment in the non-CKD group, but not in the CKD group.

**Conclusion:**

This study elucidates the associations and differences of synthetic EDs with renal function. Based on the findings, enhanced monitoring and health education regarding environmental exposure to synthetic EDs such as N-perfluorooctanoic acid, MECP phthalate, and Diethylthiophosphate can be implemented to reduce public exposure risks.

## Background

The kidney, as a crucial organ for metabolic and excretory functions in the human body, plays a vital role in maintaining systemic homeostasis. In recent years, the prevalence of Chronic Kidney Disease (CKD) and the incidence of its complications have shown a continuous upward trend, posing a significant threat to human health and quality of life (1). Recent systematic reviews by Chesnaye et al. and Francis et al. have comprehensively identified factors contributing to CKD, include age, other chronic diseases, lifestyle, and environmental pollutants (2, 3). Among these, environmental pollutants encompass both Endocrine Disruptors (EDs) such as pesticides and organophosphates, and non-EDs such as heavy metals (4, 5). EDs, which are chemical substances capable of disrupting the hormonal balance and homeostasis in organisms, can be classified into synthetic and natural EDs based on their sources (6). Synthetic EDs, such as perfluoro/polyfluoroalkyl compounds, plasticizers, and phosphorus-containing pesticides, are widely present in daily life products, including pesticides, herbicides, detergents, cosmetics, food packaging, children’s toys, and textiles, making them inevitable environmental pollutants in modern society (7). Synthetic EDs have multiple exposure routes in humans, including dietary intake, airborne inhalation, water consumption, and dermal contact. These routes increase the exposure risk and pose potential threats to human health.

Studies have shown that various synthetic EDs, such as perfluoro/polyfluoroalkyl compounds, bisphenol A, phthalates, and organophosphates, can disrupt multiple metabolic processes. These include metabolism, insulin function, stress response, and inflammation. These compounds have significantly increased the risk of various chronic diseases, including cancer, diabetes, metabolic syndrome, reproductive disorders, immune dysfunction, thyroid dysfunction, and cardiovascular diseases, many of which are associated with impaired renal function (8–12). The age-related decline in renal function and reduced metabolic clearance capacity may enhance organismal susceptibility to the toxic effects of synthetic EDs, underscoring the clinical significance of investigating the association between environmental pollutant exposure and renal impairment in middle-aged and older adult populations. Therefore, investigating the extent of organ dysfunction impacted by synthetic endocrine disruptors in middle-aged and older adult populations has become a critical research priority.

Existing research on the association between synthetic EDs and renal function has primarily focused on perfluoro/polyfluoroalkyl compounds. Epidemiological studies have shown that perfluoro/polyfluoroalkyl compounds are associated with kidney health impairment (13, 14). Stanifer et al. (15) found an association between exposure to perfluoro/polyfluoroalkyl compounds and adverse kidney outcomes, including renal function decline and kidney cancer. Furthermore, animal experiments have also demonstrated the adverse effects of perfluoro/polyfluoroalkyl compounds on kidney health, with studies revealing kidney weight gain, increased blood urea nitrogen, tubular atrophy, and tubular epithelial hyperplasia in animals exposed to these compounds (16). Although studies have shown the relationship between synthetic EDs and various chronic diseases linked to renal function impairment, research on the differential associations of plasticizers and phosphorus-containing pesticide EDs with renal function is limited. This is especially true across varying renal health states. CKD patients have impaired renal function compared to non-CKD individuals. This reduces their ability to clear exogenous and endogenous harmful substances. Therefore, in-depth exploration of the differential associations between synthetic EDs and renal function in CKD and non-CKD populations is important. It has both theoretical and applied value. Based on this, this study aims to analyze the association between synthetic EDs and renal function and their differential associations in CKD and non-CKD groups, providing an effective prevention and control strategy for clinical practice and a basis for formulating reasonable environmental protection policies.

## Materials and methods

### Study design and sample

To systematically evaluate the associations between diverse synthetic EDs and renal function indicators, this study utilized data from the National Health and Nutrition Examination Survey (NHANES) conducted between 2015 and 2018. Detailed data collection and quality control methods are available at https://www.cdc.gov/nchs/nhanes/. To effectively explore factors associated with renal dysfunction, this study enrolled participants with at least one of the following conditions: CKD, dyslipidemia, hypertension, diabetes, and hyperuricemia. We strictly excluded participants. These participants had missing data on sociodemographic characteristics, synthetic EDs characteristics, lifestyle characteristics, and health characteristics. This was done to ensure data integrity and reliability. A total of 1,199 individuals were ultimately included, with their detailed sociodemographic characteristics presented in Table 1. This study had received formal approval from the ethics committee of the National Center for Health Statistics. All participants voluntarily signed informed consent forms. They did so after fully understanding the study content. This ensured ethical compliance and participant rights protection.

**Table 1:** Characteristics of the study participants.

Characteristics	Overall (*n* = 1,199)	ACR values					
		Overall (*n* = 1,199)		CKD			
				Yes (*n* = 536)		No (*n* = 663)	
		*Z//r^^^*	*p*-value	*Z//r^^^*	*p*-value	*Z//r^^^*	*p*-value
Mean age	57 (41, 68)	−0.026	0.371	−0.059	0.171	0.030	0.438
Gender							
Male	569 (47.5)	−0.864	0.388	−4.120	<0.001***	0.231	0.818
Female	630 (52.5)						
Education level							
Less than 9th grade	94 (7.8)	3.207	0.524	3.148	0.490	4.722	0.317
9-11th grade	141 (11.8)						
High school graduate/GED or equivalent	283 (23.6)						
Some college or AA degree	386 (32.2)						
College graduate or above	295 (24.6)						
Marital status							
Married	638 (53.2)	4.725	0.450	12.814	0.025*	11.110	0.049*
Widowed	106 (8.8)						
Divorced	158 (13.2)						
Separated	47 (3.9)						
Never married	147 (12.3)						
Living with partner	103 (8.6)						
Mean family PIR	2.200 (1.190, 309.410)	−0.037	0.197	−0.112	0.009**	0.029	0.456
Mean BMI (kg/m^2^)	29.500 (25.500, 34.500)	−0.015	0.609	0.029	0.509	−0.044	0.257
Smoking status							
Smoked at least 100 cigarettes in life	548 (45.7)	−0.945	−0.141	−0.247	0.805	−0.454	0.650
Not smoked at least 100 cigarettes in life	651 (54.3)						
Physical activity level							
Reached the WHO recommended	862 (71.9)	0.706	0.916	0.630	0.529	1.124	0.261
Not reached the WHO recommended	337 (28.1)						
Dietary intakes status							
Mean milk and dairy products (g/d)	140.130 (30.000, 309.410)	−0.030	0.295	−0.029	0.497	−0.035	0.365
Mean meat and meat products (g/d)	0 (0, 18.500)	0.017	0.549	0.027	0.538	−0.002	0.960
Mean poultry and poultry products (g/d)	0 (0, 55.000)	0.040	0.169	0.053	0.222	0.032	0.415
Mean animal internal organs and products (g/d)	0 (0, 17.500)	−0.017	0.546	−0.038	0.384	0.013	0.741
Mean fish shellfish and mollusk (g/d)	0 (0, 0)	−0.002	0.939	−0.018	0.677	0.023	0.551
Mean mixed meat and vegetables (g/d)	7.500 (0, 130.000)	−0.003	0.922	−0.012	0.782	−0.018	0.651
Mean eggs and egg products (g/d)	0 (0, 30.000)	−0.022	0.448	−0.063	0.145	−0.028	0.470
Mean beans and nuts (g/d)	0 (0, 32.92)	0.042	0.143	0.067	0.123	0.029	0.462
Mean cereals and cereal products (g/d)	248.250 (129.520, 410.910)	0.011	0.697	0.004	0.934	0.002	0.966
Mean fruits and fruit products (g/d)	92.000 (0, 242.400)	−0.006	0.845	0.012	0.785	0.004	0.923
Mean vegetables and vegetable products (g/d)	70.880 (20.500, 162.530)	0.008	0.774	0.068	0.117	−0.046	0.239
Mean edible oils (g/d)	0 (0, 7.160)	0.028	0.328	0.047	0.278	0.011	0.777
Mean high-sugar processed products (g/d)	372.000 (114.000, 789.000)	0.002	0.952	0.027	0.526	−0.047	0.226
Mean water (g/d)	542.500 (204.490, 1151.250)	0.015	0.606	0.042	0.337	−0.013	0.731
Hypertension status							
Had Hypertension	786 (65.6)	−3.148	0.002**	−0.841	0.400	−0.194	0.846
Not had Hypertension	413 (34.4)						
Dyslipidemia status							
Had dyslipidemia	1,033 (86.2)	−2.780	0.005**	−0.105	0.917	−1.358	0.175
Not had dyslipidemia	166 (13.8)						
Diabetes status							
Had diabetes	528 (44.0)	4.610	<0.001***	5.315	<0.001***	2.316	0.021*
Not had diabetes	671 (56.0)						
Hyperuricemia status							
Had hyperuricemia	373 (31.1)	−0.793	0.428	−0.743	0.457	−1.524	0.127
Not had hyperuricemia	826 (68.9)						

* *p*<0.05, ** *p*<0.01, *** *p*<0.001.

^ Correlation analysis was performed using *Spearman* test. The *Mann–Whitney U* test was employed for the rank sum test between two groups, while the *Kruskal-Wallis H* test was used for the rank sum test among multiple groups.

### Measurement of synthetic endocrine disruptors

The synthetic EDs in this study included perfluoro/polyfluoroalkyl compounds, phosphorus-containing pesticides, and plasticizers, as detailed in Table 2. Perfluoro/polyfluoroalkyl compounds comprised eight types. These were Perfluorodecanoic acid (PFDE), Perfluorohexane sulfonic acid (PFHS), 2-(N-methyl-PFOSA)acetic acid (MPAH), Perfluorononanoic acid (PFNA), Perfluoroundecanoic acid (PFUA), N-perfluorooctanoic acid (NFOA), Br. perfluorooctanoic acid iso (BFOA), and N-perfluorooctane sulfonic acid (NFOS). The specific measurement methods for synthetic endocrine disruptors can be found at: https://wwwn.cdc.gov/nchs/nhanes/continuousnhanes/labmethods.aspx?BeginYear=2015&_refluxos=a10. The concentrations of perfluoro/polyfluoroalkyl compounds were determined using solid-phase extraction-high-performance liquid chromatography-electrospray ionization-mass spectrometry. Phosphorus-containing pesticides included six types. These were Dimethylphosphate (OP1), Diethylphosphate (OP2), Dimethylthiophosphate (OP3), Diethylthiophosphate (OP4), Dimethyldithiophosphate (OP5), and Diethyldithiophosphate (OP6). The concentrations of phosphorus-containing pesticides were determined using solid-phase extraction (SPE) and isotope dilution ultra-high-performance liquid chromatography-mass spectrometry. Plasticizers included 14 types. These were Mono(carboxynonyl) Phthalate (CNP), Mono(carboxyoctyl) Phthalate (COP), MECP phthalate (ECP), Mono-n-butyl phthalate (MBP), Mono-(3-carboxypropyl) phthalate (MC1), MCOCH phthalate (MCOH), Mono-ethyl phthalate (MEP), Mono-3-hydroxy-n-butyl phthalate (MHBP), MHNCH (MHNC), Mono-(2-ethyl)-hexyl phthalate (MHP), Mono-isobutyl phthalate (MIB), Mono-isononyl phthalate (MNP), MEOH phthalate (MOH), and Mono-benzyl phthalate (MZP). The concentrations of plasticizers were determined using high-performance liquid chromatography-electrospray ionization-mass spectrometry.

**Table 2:** Synthetic endocrine disruptors association with renal function under *Q10*–*Q30*.

Endocrine disruptors ^&^	*Q10* (ACR = 3.76)					*Q20* (ACR = 4.62)					*Q30* (ACR = 5.50)				
	*β*	*SE*	*t*	*p*-value	95% CI	*β*	SE	*t*	*p*-value	95% CI	*β*	SE	*t*	*p*-value	95% CI
PFDE	0.920	1.094	0.841	0.400	−1.225 ~ 3.066	0.768	0.957	0.803	0.422	−1.109 ~ 2.645	0.453	0.943	0.480	0.632	−1.398 ~ 2.304
PFHS	0.143	0.151	0.947	0.344	−0.153 ~ 0.439	0.090	0.101	0.890	0.374	−0.108 ~ 0.287	−0.003	0.113	−0.030	0.976	−0.226 ~ 0.219
MPAH	0.104	0.527	0.197	0.844	−0.930 ~ 1.138	−0.538	0.630	−0.854	0.393	−1.774 ~ 0.698	−0.387	0.666	−0.581	0.561	−1.695 ~ 0.920
PFNA	−0.065	0.410	−0.158	0.874	−0.870 ~ 0.740	0.011	0.382	0.030	0.976	−0.737 ~ 0.760	−0.053	0.426	−0.125	0.901	−0.889 ~ 0.783
PFUA	−1.383	1.946	−0.711	0.477	−5.200 ~ 2.435	−1.861	1.572	−1.184	0.237	−4.945 ~ 1.222	−2.005	1.693	−1.184	0.237	−5.327 ~ 1.318
NFOA	0.006	0.119	0.050	0.96	−0.228 ~ 0.240	0.127	0.124	1.026	0.305	−0.116 ~ 0.371	0.247	0.145	1.699	0.090	−0.038 ~ 0.532
BFOA	−10.900	7.367	−1.480	0.139	−25.354 ~ 3.555	−4.953	7.108	−0.697	0.486	−18.898 ~ 8.993	−3.816	7.257	−0.526	0.599	−18.054 ~ 10.421
NFOS	0.022	0.050	0.450	0.653	−0.076 ~ 0.121	0.047	0.039	1.209	0.227	−0.029 ~ 0.123	0.075	0.048	1.584	0.114	−0.018 ~ 0.169
OP1	0.004	0.021	0.211	0.833	−0.036 ~ 0.045	0.004	0.017	0.252	0.801	−0.029 ~ 0.037	0.001	0.015	0.086	0.932	−0.028 ~ 0.030
OP2	−0.005	0.006	−0.822	0.411	−0.017 ~ 0.007	0.001	0.007	0.169	0.866	−0.013 ~ 0.015	−0.010	0.010	−1.030	0.303	−0.029 ~ 0.009
OP3	−0.019	0.032	−0.593	0.553	−0.083 ~ 0.044	−0.002	0.038	−0.046	0.963	−0.077 ~ 0.073	0.014	0.038	0.372	0.710	−0.060 ~ 0.088
OP4	0.109	0.069	1.567	0.117	−0.027 ~ 0.245	0.222	0.076	2.933	0.003**	0.073 ~ 0.370	0.326	0.089	3.684	<0.001***	0.153 ~ 0.500
OP5	0.027	0.118	0.225	0.822	−0.205 ~ 0.258	−0.011	0.140	−0.078	0.937	−0.285 ~ 0.263	−0.082	0.128	−0.643	0.52	−0.334 ~ 0.169
OP6	1.600	5.128	0.312	0.755	−8.461 ~ 11.660	4.613	4.563	1.011	0.312	−4.341 ~ 13.566	3.325	5.581	0.596	0.551	−7.625 ~ 14.275
CNP	−0.023	0.029	−0.790	0.43	−0.081 ~ 0.034	−0.033	0.026	−1.259	0.208	−0.085 ~ 0.019	−0.045	0.028	−1.605	0.109	−0.100 ~ 0.010
COP	−0.001	0.006	−0.232	0.817	−0.014 ~ 0.011	0.000	0.006	0.083	0.934	−0.011 ~ 0.012	−0.003	0.006	−0.526	0.599	−0.015 ~ 0.009
ECP	0.026	0.004	7.352	<0.001***	0.019 ~ 0.033	0.012	0.005	2.411	0.016*	0.002 ~ 0.023	0.028	0.007	4.074	<0.001***	0.015 ~ 0.042
MBP	−0.005	0.015	−0.318	0.750	−0.035 ~ 0.025	−0.006	0.017	−0.354	0.724	−0.039 ~ 0.027	−0.017	0.015	−1.166	0.244	−0.046 ~ 0.012
MC1	0.000	0.067	0.006	0.995	−0.132 ~ 0.133	−0.002	0.056	−0.042	0.966	−0.112 ~ 0.107	−0.012	0.051	−0.231	0.817	−0.111 ~ 0.088
MCOH	0.026	0.182	0.143	0.886	−0.330 ~ 0.382	0.011	0.149	0.071	0.943	−0.282 ~ 0.304	−0.044	0.098	−0.449	0.654	−0.236 ~ 0.148
MEP	0.000	0.000	0.166	0.868	−0.001 ~ 0.001	0.000	0.000	−0.196	0.845	−0.001 ~ 0.001	0.000	0.000	−0.596	0.551	−0.001 ~ <0.001***
MHBP	0.013	0.151	0.084	0.933	−0.284 ~ 0.310	0.029	0.183	0.159	0.873	−0.330 ~ 0.388	0.299	0.173	1.725	0.085	−0.041 ~ 0.639
MHNC	0.009	0.066	0.137	0.891	−0.121 ~ 0.139	0.014	0.059	0.231	0.817	−0.102 ~ 0.129	0.050	0.052	0.956	0.339	−0.052 ~ 0.152
MHP	0.037	0.056	0.671	0.502	−0.072 ~ 0.146	−0.001	0.065	−0.020	0.984	−0.129 ~ 0.126	−0.013	0.070	−0.185	0.854	−0.150 ~ 0.124
MIB	0.012	0.028	0.429	0.668	−0.043 ~ 0.067	0.012	0.024	0.514	0.607	−0.035 ~ 0.060	0.017	0.017	1.017	0.309	−0.016 ~ 0.050
MNP	−0.001	0.054	−0.015	0.988	−0.106 ~ 0.104	0.000	0.060	0.004	0.996	−0.117 ~ 0.118	0.058	0.066	0.884	0.377	−0.071 ~ 0.188
MOH	0.070	0.055	1.277	0.202	−0.038 ~ 0.177	0.054	0.061	0.881	0.379	−0.066 ~ 0.175	−0.003	0.070	−0.036	0.971	−0.139 ~ 0.134
MZP	<0.001	0.003	0.096	0.923	−0.005 ~ 0.006	<0.001	0.003	0.134	0.893	−0.006 ~ 0.007	0.001	0.003	0.218	0.828	−0.006 ~ 0.008

^&^ PFDE, Perfluorodecanoic acid (ng/mL); PFHS, Perfluorohexane sulfonic acid (ng/mL); MPAH, 2-(N-methyl-PFOSA)acetic acid (ng/mL); PFNA, Perfluorononanoic acid (ng/mL); PFUA, Perfluoroundecanoic acid (ng/mL); NFOA, N-perfluorooctanoic acid (ng/mL); BFOA, Br. perfluorooctanoic acid iso (ng/mL); NFOS, N-perfluorooctane sulfonic acid (ng/mL); OP1, Dimethylphosphate (ng/mL); OP2, Diethylphosphate (ng/mL); OP3, Dimethylthiophosphate (ng/mL); OP4, Diethylthiophosphate (ng/mL); OP5, Dimethyldithiophosphate (ng/mL); OP6, Diethyldithiophosphate (ng/mL); CNP, Mono(carboxynonyl) Phthalate (ng/mL); COP, Mono(carboxyoctyl) Phthalate (ng/mL); ECP, MECP phthalate (ng/mL); MBP, Mono-n-butyl phthalate (ng/mL); MC1, Mono-(3-carboxypropyl) phthalate (ng/mL); MCOH, MCOCH phthalate (ng/mL); MEP, Mono-ethyl phthalate (ng/mL); MHBP, Mono-3-hydroxy-n-butyl phthalate (ng/mL); MHNC, MHNCH (ng/mL); MHP, Mono-(2-ethyl)-hexyl phthalate (ng/mL); MIB, Mono-isobutyl phthalate (ng/mL); MNP, Mono-isononyl phthalate (ng/mL); MOH, MEOH phthalate (ng/mL); MZP, Mono-benzyl phthalate (ng/mL). Q (Quantile) represents the number of categories; β is the standardized regression coefficient; SE (Standard Error) is the standard error, indicating the fluctuation of the B value; t is the t-test statistic used to test the significance of the regression coefficient; *p* is used to determine whether the analysis item shows significance, with a p-value less than 0.05 considered significant; 95% CI represents the 95% confidence interval.

**Table 3:** Synthetic endocrine disruptors association with renal function under *Q40*–*Q60*.

Endocrine disruptors ^&^	*Q40* (ACR = 6.71)					*Q50* (ACR = 8.16)					*Q60* (ACR = 10.650)				
	*β*	SE	*t*	*p*-value	95% CI	*β*	SE	*t*	*p*-value	95% CI	*β*	SE	*t*	*p*-value	95% CI
PFDE	0.465	1.048	0.443	0.658	−1.592 ~ 2.521	0.402	1.244	0.324	0.746	−2.038 ~ 2.842	0.843	1.701	0.496	0.620	−2.494 ~ 4.179
PFHS	−0.008	0.151	−0.055	0.956	−0.305 ~ 0.289	0.134	0.180	0.742	0.458	−0.220 ~ 0.488	0.421	0.251	1.676	0.094	−0.072 ~ 0.913
MPAH	0.520	0.751	0.692	0.489	−0.954 ~ 1.994	0.586	0.943	0.622	0.534	−1.264 ~ 2.437	−0.063	1.426	−0.044	0.965	−2.860 ~ 2.734
PFNA	−0.502	0.497	−1.011	0.312	−1.476 ~ 0.472	−1.037	0.596	−1.739	0.082	−2.207 ~ 0.133	−1.331	0.841	−1.582	0.114	−2.981 ~ 0.319
PFUA	−3.102	1.910	−1.624	0.105	−6.851 ~ 0.646	−1.378	2.301	−0.599	0.549	−5.894 ~ 3.137	−4.303	3.213	−1.339	0.181	−10.607 ~ 2.002
NFOA	0.724	0.187	3.865	<0.001***	0.356 ~ 1.092	1.157	0.224	5.160	<0.001***	0.717 ~ 1.597	1.108	0.313	3.538	<0.001***	0.494 ~ 1.723
BFOA	4.544	8.365	0.543	0.587	−11.869 ~ 20.956	−1.826	10.811	−0.169	0.866	−23.036 ~ 19.385	−4.408	15.081	−0.292	0.770	−33.996 ~ 25.180
NFOS	0.123	0.057	2.158	0.031*	0.011 ~ 0.235	0.107	0.067	1.598	0.110	−0.024 ~ 0.239	0.211	0.096	2.206	0.028*	0.023 ~ 0.398
OP1	0.004	0.014	0.298	0.765	−0.024 ~ 0.033	0.006	0.016	0.355	0.722	−0.026 ~ 0.037	0.007	0.025	0.256	0.798	−0.043 ~ 0.056
OP2	−0.011	0.012	−0.850	0.395	−0.035 ~ 0.014	−0.012	0.017	−0.707	0.480	−0.045 ~ 0.021	−0.021	0.027	−0.773	0.440	−0.074 ~ 0.032
OP3	0.026	0.045	0.565	0.572	−0.063 ~ 0.115	0.041	0.054	0.768	0.443	−0.064 ~ 0.147	−0.020	0.077	−0.252	0.801	−0.172 ~ 0.132
OP4	0.331	0.118	2.807	0.005**	0.100 ~ 0.562	0.928	0.143	6.496	<0.001***	0.648 ~ 1.208	2.210	0.213	10.397	<0.001***	1.793 ~ 2.627
OP5	−0.185	0.170	−1.085	0.278	−0.518 ~ 0.149	−0.353	0.204	−1.729	0.084	−0.754 ~ 0.048	−0.261	0.298	−0.876	0.381	−0.846 ~ 0.324
OP6	6.676	5.839	1.143	0.253	−4.780 ~ 18.132	0.040	6.903	0.006	0.995	−13.503 ~ 13.583	−7.977	8.979	−0.888	0.375	−25.594 ~ 9.640
CNP	−0.035	0.032	−1.092	0.275	−0.098 ~ 0.028	−0.031	0.041	−0.753	0.452	−0.111 ~ 0.050	0.053	0.059	0.903	0.367	−0.062 ~ 0.168
COP	0.000	0.007	0.033	0.973	−0.013 ~ 0.013	0.009	0.008	1.108	0.268	−0.007 ~ 0.025	0.006	0.012	0.496	0.620	−0.017 ~ 0.029
ECP	0.027	0.009	2.872	0.004**	0.009 ~ 0.046	0.025	0.014	1.834	0.067	−0.002 ~ 0.052	0.021	0.023	0.897	0.370	−0.025 ~ 0.067
MBP	−0.008	0.015	−0.502	0.616	−0.038 ~ 0.022	−0.026	0.018	−1.461	0.144	−0.060 ~ 0.009	−0.025	0.023	−1.114	0.266	−0.069 ~ 0.019
MC1	−0.030	0.050	−0.592	0.554	−0.129 ~ 0.069	−0.042	0.056	−0.762	0.446	−0.151 ~ 0.067	−0.005	0.075	−0.063	0.950	−0.151 ~ 0.142
MCOH	−0.095	0.116	−0.823	0.411	−0.323 ~ 0.132	−0.092	0.155	−0.594	0.553	−0.396 ~ 0.212	−0.074	0.241	−0.308	0.758	−0.547 ~ 0.398
MEP	0.000	0.000	−1.014	0.311	−0.001 ~ <0.001***	0.000	0.000	−0.899	0.369	−0.001 ~ 0.001	−0.001	0.001	−1.366	0.172	−0.002 ~ <0.001***
MHBP	0.178	0.185	0.960	0.337	−0.185 ~ 0.540	0.398	0.218	1.831	0.067	−0.028 ~ 0.825	0.416	0.294	1.412	0.158	−0.162 ~ 0.993
MHNC	0.080	0.059	1.366	0.172	−0.035 ~ 0.196	0.065	0.076	0.852	0.394	−0.085 ~ 0.215	0.035	0.113	0.309	0.758	−0.187 ~ 0.257
MHP	0.043	0.083	0.514	0.607	−0.120 ~ 0.205	0.091	0.109	0.837	0.403	−0.122 ~ 0.304	0.119	0.167	0.713	0.476	−0.209 ~ 0.447
MIB	0.017	0.020	0.855	0.393	−0.023 ~ 0.057	0.023	0.026	0.858	0.391	−0.029 ~ 0.074	0.061	0.036	1.725	0.085	−0.008 ~ 0.131
MNP	0.055	0.073	0.761	0.447	−0.087 ~ 0.198	−0.005	0.082	−0.061	0.952	−0.166 ~ 0.156	−0.047	0.117	−0.399	0.690	−0.276 ~ 0.183
MOH	0.076	0.084	0.904	0.366	−0.088 ~ 0.240	0.156	0.110	1.426	0.154	−0.059 ~ 0.371	0.227	0.167	1.358	0.175	−0.101 ~ 0.554
MZP	0.004	0.004	0.874	0.382	−0.005 ~ 0.012	0.004	0.005	0.771	0.441	−0.006 ~ 0.015	0.002	0.008	0.223	0.823	−0.014 ~ 0.018

Q (Quantile) represents the number of categories; β is the standardized regression coefficient; SE (Standard Error) is the standard error, indicating the fluctuation of the B value; t is the t-test statistic used to test the significance of the regression coefficient; p is used to determine whether the analysis item shows significance, with a p-value less than 0.05 considered significant; 95% CI represents the 95% confidence interval.

### Measurement of renal function and CKD

Currently, three well-validated biomarkers are widely used to evaluate renal function, including the estimated glomerular filtration rate (eGFR), albumin-to-creatinine ratio (ACR), and total protein-to-creatinine ratio (PCR). Among these, ACR is more advantageous in the early diagnosis of CKD due to its high sensitivity, convenience, and low cost. Therefore, considering its advantages, this study employed ACR to assess renal function. CKD was defined according to established clinical criteria. Participants needed to fulfill at least one of the following conditions: (1) persistent renal dysfunction (abnormal kidney structure or renal function) for more than 3 months; (2) ACR ≥ 30 mg/g; (3) glomerular filtration rate (eGFR) < 60 mL·min^−1^·(1.73 m^2^)^−1^ for more than 3 months (17–19). ACR was estimated by calculating the ratio of urine albumin to urine creatinine. eGFR was estimated using the MDRD formula based on serum creatinine (17). Urine creatinine was measured using the Roche Cobas 6,000 analyzer with an enzymatic method. Urine albumin was measured using the Sequoia-Turne 450 digital fluorometer with a fluorescence immunoassay. Serum creatinine was measured using the Beckman UniCel DxC 800 Synchron, Beckman UniCel DxC 660i Synchron with the alkaline picrate Jaffe rate method or the Roche Cobas 6,000 with an enzymatic method. The specific process is detailed at https://www.cdc.gov/nchs/nhanes/.

To comprehensively assess the association between synthetic endocrine disruptors and renal function, this study employed quantile regression analysis to examine their associations across different percentiles of the exposure distribution. In contrast to conventional linear regression that estimates average effects, quantile regression minimizes weighted absolute residuals to characterize relationships at specific points of the conditional distribution. This approach provides robust estimation of covariate effects across the entire conditional distribution, particularly at specified quantiles. We specifically analyzed the associations at the following quantiles: *Q10*, *Q20*, *Q30*, *Q40*, *Q50*, *Q60*, *Q70*, *Q80*, and *Q90*. This was done to capture potential differential effects across low, moderate, and high exposure levels.

### Covariates

The control variables in this study included sociodemographic characteristics, lifestyle characteristics, and other health characteristics. Sociodemographic characteristics encompassed age, gender, marital status, education level, and family poverty-to-income ratio (PIR, i.e., the ratio of family income to the poverty line). Lifestyle characteristics included smoking status (whether smoking more than 100 cigarettes). They also included physical activity level, which was assessed based on the WHO-recommended Global Physical Activity Questionnaire guidelines. The threshold was 150 min of moderate-intensity activity, 75 min of high-intensity activity, or 600 metabolic equivalents (METs) per week. Additionally, they included dietary intake status, which assessed daily intake of nine types of food. This study also assessed body mass index (BMI), as well as the presence of dyslipidemia, hypertension, diabetes, and hyperuricemia as other health characteristics. Dyslipidemia was defined as fulfillment of at least one of the following conditions: (1) hypercholesterolemia (total cholesterol ≥ 5.18 mmol/L); (2) hypertriglyceridemia (triglycerides ≥ 1.70 mmol/L); (3) low-density lipoprotein cholesterol (LDL-C) ≥ 3.37 mmol/L; (4) high-density lipoprotein cholesterol (HDL-C) < 1.04 mmol/L (20). Hypertension was defined as an average systolic blood pressure > 140 mmHg or an average diastolic blood pressure > 90 mmHg, or a physician diagnosis of hypertension (21). Diabetes was defined as a fasting blood glucose level ≥ 7.0 mmol/L or a 2-h postprandial blood glucose level ≥ 11.1 mmol/L, or a physician diagnosis of diabetes (22, 23). Hyperuricemia was defined as a serum uric acid level above 420 μmol/L (7 mg/dL) for males and above 357 μmol/L (6 mg/dL) for females (24). These characteristics are detailed in Table 1.

### Statistical analysis

For continuous variables exhibiting non-normal distributions, such as ACR, age, PIR, BMI, and dietary intake, *P_50_* (*P_25_*, *P_75_*) was used for statistical descriptive analysis. For categorical variables, including gender, marital status, education level, smoking status, physical activity level, and the presence of dyslipidemia, hypertension, diabetes, and hyperuricemia, frequency (proportion) was used for statistical descriptive analysis. To determine the differences in ACR distribution across sociodemographic characteristics, *Spearman* tests, *Mann–Whitney U* tests, and *Kruskal-Wallis* tests were employed. Spearman tests were used for correlation analysis. *Mann–Whitney U* tests were used for rank sum tests between two groups, and Kruskal-Wallis H tests were used for rank sum tests among multiple groups. After controlling for sociodemographic, lifestyle, and other health characteristics, quantile regression models were used to explore the association between synthetic EDs and renal function, and grouped regression models were used to analyze the differential association between synthetic EDs and renal function in CKD and non-CKD groups. All continuous variables were transformed for normality before being included in the regression models. All tests were based on two-sided tests with a 95% confidence interval.

## Results

### Participant characteristics

The basic characteristics of the participants are shown in Table 1. About half were female (52.5%), 24.6% had a college or associate degree or higher, 53.2% were married, 45.7% had smoked at least 100 cigarettes in their lifetime, and 71.9% met the WHO-recommended physical activity level. The median age was 57 years (*P_50_*), with *P_25_* at 41 and *P_75_* at 68 years. The median PIR was 2.200 (*P_50_*), with *P_25_* at 1.190 and *P_75_* at 309.410. Additionally, the median BMI was 29.5 (*P_50_*), with *P_25_* at 25.5 and *P_75_* at 34.5. Over half had hypertension (65.6%), 86.2% had dyslipidemia, 44.0% had diabetes, 31.1% had hyperuricemia, and 44.7% had CKD. The median ACR was 8.160 (*P_50_*), with *P_25_* at 5.040 and *P_75_* at 20.170.

Table 1 provides a detailed distribution of ACR values among participants. The study demonstrated significant correlations between the prevalence of hypertension, dyslipidemia, and diabetes mellitus and renal function decline, as well as ACR values. The *Z*-values were −3.148, −2.780, and 4.610, respectively (*p <* 0.01). In the CKD group, multivariate analysis revealed significant associations between gender, marital status, family PIR, diabetes status, and ACR values (*p <* 0.05). In non-CKD participants, only marital status and diabetes were significantly associated with ACR levels (*p* < 0.05).

### Association between synthetic endocrine disruptors and renal function

The comprehensive association between synthetic EDs and renal function is presented in Tables 2–4. Tables 2–4 show that N-perfluorooctanoic acid and N-perfluorooctane sulfonic acid among perfluoro/polyfluoroalkyl compounds, Diethylthiophosphate among phosphorus-containing pesticides, and MECP phthalate, Mono-isobutyl phthalate, Mono-isononyl phthalate, and MEOH phthalate among plasticizers were associated with renal function impairment (*p <* 0.05).

**Table 4:** Synthetic endocrine disruptors association with renal function under *Q70*–*Q90*.

Endocrine disruptors ^&^	*Q70* (ACR = 15.570)					*Q80* (ACR = 29.01)					*Q90* (ACR = 80.00)				
	*β*	SE	*t*	*p*-value	95% CI	*β*	SE	*t*	*p*-value	95% CI	*β*	SE	*t*	*p*-value	95% CI
PFDE	−2.616	3.171	−0.825	0.410	−8.838 ~ 3.606	−1.529	7.421	−0.206	0.837	−16.090 ~ 13.031	4.449	15.912	0.280	0.780	−26.771 ~ 35.669
PFHS	0.741	0.465	1.596	0.111	−0.170 ~ 1.653	1.275	1.078	1.183	0.237	−0.841 ~ 3.391	2.006	2.124	0.945	0.345	−2.160 ~ 6.173
MPAH	−1.074	2.594	−0.414	0.679	−6.163 ~ 4.016	−4.278	6.841	−0.625	0.532	−17.700 ~ 9.143	−6.379	16.229	−0.393	0.694	−38.221 ~ 25.463
PFNA	−2.676	1.708	−1.567	0.117	−6.028 ~ 0.675	−4.913	4.084	−1.203	0.229	−12.925 ~ 3.099	−19.144	9.857	−1.942	0.052	−38.483 ~ 0.195
PFUA	−0.413	6.040	−0.068	0.946	−12.263 ~ 11.438	14.721	14.719	1.000	0.317	−14.157 ~ 43.599	39.630	30.944	1.281	0.201	−21.082 ~ 100.342
NFOA	1.511	0.593	2.550	0.011*	0.348 ~ 2.673	5.074	1.354	3.747	<0.001***	2.417 ~ 7.732	9.579	2.451	3.909	<0.001***	4.771 ~ 14.387
BFOA	−20.693	28.286	−0.732	0.465	−76.191 ~ 34.804	2.714	66.357	0.041	0.967	−127.477 ~ 132.906	183.551	155.218	1.183	0.237	−120.987 ~ 488.089
NFOS	0.389	0.188	2.068	0.039*	0.020 ~ 0.757	−0.127	0.523	−0.244	0.808	−1.152 ~ 0.898	0.618	0.842	0.734	0.463	−1.034 ~ 2.270
OP1	0.000	0.057	−0.005	0.996	−0.112 ~ 0.111	0.015	0.165	0.088	0.930	−0.310 ~ 0.339	−0.027	0.453	−0.060	0.952	−0.916 ~ 0.862
OP2	−0.047	0.053	−0.903	0.367	−0.151 ~ 0.056	−0.098	0.151	−0.652	0.514	−0.395 ~ 0.198	−0.263	0.297	−0.886	0.376	−0.845 ~ 0.320
OP3	−0.026	0.154	−0.168	0.866	−0.327 ~ 0.276	−0.278	0.342	−0.813	0.416	−0.949 ~ 0.393	−0.515	0.788	−0.654	0.513	−2.062 ~ 1.031
OP4	5.858	0.441	13.287	<0.001***	4.993 ~ 6.723	10.417	1.013	10.280	<0.001***	8.429 ~ 12.405	63.045	2.550	24.726	<0.001***	58.042 ~ 68.047
OP5	−0.505	0.618	−0.818	0.413	−1.717 ~ 0.707	−0.151	1.609	−0.094	0.925	−3.308 ~ 3.006	−2.493	3.922	−0.636	0.525	−10.188 ~ 5.201
OP6	−19.891	15.759	−1.262	0.207	−50.810 ~ 11.027	−18.248	35.732	−0.511	0.610	−88.355 ~ 51.859	−128.432	67.322	−1.908	0.057	−260.518 ~ 3.654
CNP	0.142	0.114	1.240	0.215	−0.082 ~ 0.366	0.045	0.305	0.147	0.883	−0.554 ~ 0.644	−0.604	0.469	−1.289	0.198	−1.524 ~ 0.316
COP	−0.015	0.021	−0.693	0.488	−0.056 ~ 0.027	−0.036	0.057	−0.634	0.526	−0.147 ~ 0.075	−0.101	0.096	−1.046	0.296	−0.289 ~ 0.088
ECP	0.007	0.054	0.123	0.902	−0.099 ~ 0.112	−0.018	0.079	−0.235	0.814	−0.173 ~ 0.136	−0.120	0.434	−0.277	0.782	−0.972 ~ 0.732
MBP	−0.017	0.039	−0.432	0.666	−0.093 ~ 0.060	−0.059	0.118	−0.496	0.620	−0.290 ~ 0.173	−0.082	0.244	−0.336	0.737	−0.561 ~ 0.397
MC1	−0.072	0.117	−0.619	0.536	−0.302 ~ 0.157	−0.186	0.263	−0.707	0.480	−0.703 ~ 0.331	−0.455	0.427	−1.066	0.287	−1.293 ~ 0.382
MCOH	−0.224	0.528	−0.425	0.671	−1.260 ~ 0.811	−1.000	1.538	−0.650	0.516	−4.017 ~ 2.017	−1.050	1.498	−0.701	0.483	−3.989 ~ 1.889
MEP	−0.001	0.001	−1.131	0.258	−0.004 ~ 0.001	−0.004	0.003	−1.392	0.164	−0.010 ~ 0.002	−0.009	0.008	−1.161	0.246	−0.024 ~ 0.006
MHBP	0.320	0.548	0.584	0.559	−0.756 ~ 1.396	0.566	1.389	0.408	0.683	−2.158 ~ 3.291	−0.418	3.135	−0.133	0.894	−6.568 ~ 5.733
MHNC	0.105	0.237	0.445	0.657	−0.360 ~ 0.571	0.585	0.666	0.878	0.380	−0.722 ~ 1.891	0.277	1.017	0.273	0.785	−1.718 ~ 2.273
MHP	0.016	0.352	0.044	0.965	−0.674 ~ 0.705	0.065	0.634	0.102	0.919	−1.180 ~ 1.309	−1.054	0.983	−1.071	0.284	−2.983 ~ 0.876
MIB	0.176	0.066	2.670	0.008**	0.047 ~ 0.306	0.383	0.164	2.332	0.020*	0.061 ~ 0.705	0.581	0.299	1.942	0.052	−0.006 ~ 1.168
MNP	0.180	0.200	0.903	0.367	−0.211 ~ 0.572	0.437	0.483	0.903	0.367	−0.512 ~ 1.385	3.585	0.893	4.014	<0.001***	1.833 ~ 5.338
MOH	0.723	0.352	2.056	0.040*	0.033 ~ 1.414	1.411	0.790	1.786	0.074	−0.139 ~ 2.961	0.887	1.881	0.472	0.637	−2.804 ~ 4.579
MZP	−0.006	0.018	−0.337	0.736	−0.041 ~ 0.029	−0.019	0.048	−0.386	0.700	−0.113 ~ 0.076	−0.010	0.071	−0.142	0.887	−0.150 ~ 0.130

Q (Quantile) represents the number of categories; β is the standardized regression coefficient; SE (Standard Error) is the standard error, indicating the fluctuation of the B value; t is the t-test statistic used to test the significance of the regression coefficient; p is used to determine whether the analysis item shows significance, with a p-value less than 0.05 considered significant; 95% CI represents the 95% confidence interval.

N-perfluorooctanoic acid was associated with renal function impairment when ACR values were at *Q40* or above (*p* < 0.05). The damaging effect increased with increasing ACR values. Specifically, at ACR *Q40*, *β* = 0.724 (95% CI: 0.356, 1.092). At ACR *Q50*, *β* = 1.157 (95% CI: 0.717, 1.597). At ACR *Q60*, *β* = 1.108 (95% CI: 0.494, 1.723). At ACR *Q70*, *β* = 1.511 (95% CI: 0.348, 2.673). At ACR *Q80*, *β* = 5.074 (95% CI: 2.417, 7.732). At ACR *Q90*, *β* = 9.579 (95% CI: 4.771, 14.387). Diethylthiophosphate was also associated with renal function impairment when ACR values were above *Q20* (*p <* 0.05). The damaging effect increased with increasing ACR values. Specifically, at ACR *Q20*, *β* = 0.222 (95% CI: 0.073, 0.370). At ACR *Q30*, *β* = 0.326 (95% CI: 0.153, 0.500). At ACR *Q40*, *β* = 0.331 (95% CI: 0.100, 0.562). At ACR *Q50*, *β* = 0.928 (95% CI: 0.648, 1.208). At ACR *Q60*, *β* = 2.210 (95% CI: 1.793, 2.627). At ACR *Q70, β* = 5.858 (95% CI: 4.993, 6.723). At ACR *Q80*, *β* = 10.417 (95% CI: 8.429, 12.405). And at ACR *Q90*, *β* = 63.045 (95% CI: 58.042, 68.047). Both N-perfluorooctanoic acid and Diethylthiophosphate exhibited gradually increasing damaging effects on renal function with increasing ACR values.

MECP phthalate was associated with renal function impairment when ACR values were below *Q40* (*p <* 0.05). Specifically, at ACR *Q10*, *β* = 0.026 (95% CI: 0.019, 0.033). At ACR *Q20*, *β* = 0.012 (95% CI: 0.002, 0.023). At ACR *Q30*, *β* = 0.028 (95% CI: 0.015, 0.042). At ACR *Q40*, *β* = 0.027 (95% CI: 0.009, 0.046).

Additionally, N-perfluorooctane sulfonic acid was associated with renal function impairment in individuals with ACR values at *Q40*, *Q60*, and *Q70* (*p* < 0.05). Specifically, at ACR *Q40*, *β* = 0.123 (95% CI: 0.011, 0.235). At ACR *Q60*, *β* = 0.211 (95% CI: −0.023, 0.398). And at ACR *Q70*, *β* = 0.389 (95% CI: 0.020, 0.757). Mono-isobutyl phthalate was associated with renal function impairment in individuals with ACR values at *Q70* and *Q80* (p < 0.05). Specifically, at ACR *Q70*, *β* = 0.176 (95% CI: 0.047, 0.306). At ACR *Q80*, *β* = 0.383 (95% CI: 0.061, 0.705). Mono-isononyl phthalate was associated with renal function impairment in individuals with ACR values at *Q90* (*p* < 0.05), with *β* = 3.585 (95% CI: 1.833, 5.338). MEOH phthalate was associated with renal function impairment in individuals with ACR values at *Q70* (*p <* 0.05), with *β* = 0.723 (95% CI: 0.033, 1.414).

The experimental results of this study demonstrate that certain per- and polyfluoroalkyl substances (PFAS) and multiple plasticizers can adversely affect renal function. PFAS may impair kidney function partly because of their high chemical stability, which facilitates accumulation within renal tissues. This bioaccumulation can disrupt critical signaling pathways and metabolic processes, such as those involving fatty acid-binding proteins, peroxisome proliferator-activated receptors (PPARs) and estrogen receptors. Plasticizers, which are known to cause endocrine disruption and hepatotoxicity while potentially promoting diabetes and hyperuricemia, may indirectly influence renal function through complex physiological mechanisms. Additionally, some organophosphate pesticides were found to be nephrotoxic, likely due to their effects on cholinergic signaling, membrane toxicity, and pro-oxidative pathways, all of which may contribute to renal dysfunction.

### Differential association between synthetic endocrine disruptors and renal function: non-CKD vs. CKD

This study further explored the differential association between synthetic EDs and renal function in non-CKD and CKD groups. The details are shown in Table 5. The association between synthetic EDs and renal function differed between non-CKD and CKD groups, with Chow Test results showing *F* = 3.258, *p* < 0.001. As shown in Table 5, N-perfluorooctanoic acid had different associations with renal function in non-CKD and CKD groups. The difference was *B_1_*-*B_2_* = −3.307, *p* = 0.004. Specifically, in the non-CKD group, N-perfluorooctanoic acid showed no association with renal function impairment in the non-CKD group (*β* = 0.158, *p* = 0.029). However, in the CKD group, it was associated with impairment in the CKD group (*β* = 0.007, *p* = 0.899). MECP phthalate also had a differential association with renal function in non-CKD and CKD groups. The difference was *B_1_*-*B_2_* = 0.176, *p* = 0.002. Specifically, in the non-CKD group, MECP phthalate was associated with renal function impairment in the non-CKD group (*β* = 0.163, *p* = 0.006). In the CKD group, it was not associated with renal function (*β* = −0.024, *p* = 0.852). The damaging effects of N-perfluorooctanoic acid in perfluoro/polyfluoroalkyl compounds and diethylthiophosphate in organophosphate pesticides on renal function exhibit a progressively increasing trend. In contrast, MECP phthalate from plasticizers primarily exerts certain negative effects on individuals whose renal function has not yet suffered severe impairment. N-perfluorooctanoic acid is especially harmful to the renal function of chronic CKD patients. In contrast, MECP phthalate more readily impairs the renal function of non-CKD individuals.

**Table 5:** Synthetic endocrine disruptors association with renal function: non-CKD vs. CKD differences.

ACR																		
Endocrine disruptors ^&^	Overall					CKD STATUS												
						No					Yes							
	*B*	SE	*t*	*p*-value	*β*	*B_1_*	SE	*t*	*p*-value	*β*	*B_2_*	SE	*t*	*p*-value	*β*	*B_1_*-*B_2_*	*t*	*p*-value
PFDE	1.762	5.087	0.346	0.729	0.023	2.183	6.648	0.328	0.743	0.026	−1.579	7.644	−0.207	0.836	−0.022	3.762	0.842	0.400
PFHS	−0.937	0.738	−1.268	0.205	−0.045	−0.316	0.773	−0.409	0.683	−0.019	−2.571	1.394	−1.844	0.066	−0.105	2.255	1.947	0.052
MPAH	2.832	3.855	0.735	0.463	0.022	−1.598	5.010	−0.319	0.750	−0.013	5.965	5.719	1.043	0.297	0.047	−7.564	−1.064	0.288
PFNA	0.105	2.438	0.043	0.966	0.002	−1.318	2.882	−0.457	0.648	−0.027	0.678	3.971	0.171	0.865	0.013	−1.996	−0.690	0.490
PFUA	−12.180	9.408	−1.295	0.196	−0.079	6.679	12.393	0.539	0.590	0.041	−17.285	14.148	−1.222	0.222	−0.120	23.964	2.690	0.007**
NFOA	1.201	0.918	1.309	0.191	0.057	0.132	1.041	0.127	0.899	0.007	3.439*	1.573	2.186	0.029*	0.158	−3.307	−2.860	0.004**
BFOA	39.014	44.289	0.881	0.379	0.026	21.752	63.673	0.342	0.733	0.014	72.384	62.046	1.167	0.244	0.053	−50.632	−0.587	0.557
NFOS	0.346	0.275	1.261	0.208	0.066	−0.108	0.338	−0.319	0.750	−0.023	0.787	0.431	1.825	0.069	0.146	−0.895	−3.126	0.002**
OP1	0.035	0.064	0.552	0.581	0.018	0.045	0.059	0.752	0.452	0.035	−0.222	0.264	−0.839	0.402	−0.044	0.266	1.385	0.166
OP2	−0.033	0.069	−0.479	0.632	−0.014	−0.032	0.061	−0.520	0.603	−0.020	−0.241	0.237	−1.016	0.310	−0.048	0.209	1.087	0.277
OP3	−0.047	0.216	−0.217	0.828	−0.013	−0.288	0.223	−1.288	0.198	−0.100	0.795	0.524	1.517	0.130	0.168	−1.082	−5.090	<0.001**
OP4	−0.338	0.583	−0.580	0.562	−0.018	1.020	1.079	0.945	0.345	0.040	−0.113	0.788	−0.143	0.886	−0.007	1.133	0.884	0.377
OP5	0.409	0.827	0.495	0.621	0.028	0.851	1.048	0.812	0.417	0.060	−1.451	1.540	−0.942	0.347	−0.099	2.302	2.822	0.005**
OP6	−8.097	28.299	−0.286	0.775	−0.008	−7.767	26.974	−0.288	0.773	−0.011	−24.817	67.078	−0.370	0.712	−0.016	17.050	0.270	0.787
CNP	−0.152	0.168	−0.907	0.364	−0.028	0.142	0.238	0.596	0.552	0.025	−0.233	0.241	−0.965	0.335	−0.046	0.374	1.214	0.225
COP	−0.002	0.033	−0.066	0.947	−0.003	−0.017	0.041	−0.419	0.675	−0.035	−0.015	0.059	−0.262	0.794	−0.020	−0.002	−0.046	0.963
ECP	0.122*	0.056	2.169	0.030*	0.118	0.150**	0.055	2.738	0.006**	0.163	−0.025	0.137	−0.187	0.852	−0.024	0.176	3.117	0.002**
MBP	0.016	0.072	0.216	0.829	0.015	0.011	0.078	0.147	0.883	0.014	0.013	0.141	0.092	0.927	0.010	−0.002	−0.027	0.979
MC1	0.137	0.222	0.618	0.536	0.029	0.014	0.209	0.067	0.947	0.004	0.194	0.765	0.253	0.800	0.021	−0.180	−0.494	0.621
MCOH	−0.161	0.633	−0.255	0.799	−0.020	−0.795	0.690	−1.152	0.250	−0.119	−0.918	1.645	−0.558	0.577	−0.097	0.124	0.273	0.785
MEP	−0.002	0.001	−1.152	0.250	−0.034	<0.001	0.002	−0.308	0.759	−0.013	−0.005*	0.002	−1.983	0.048*	−0.086	0.004	1.659	0.097
MHBP	−0.661	0.890	−0.742	0.458	−0.054	−0.471	1.067	−0.441	0.659	−0.046	−0.499	1.541	−0.323	0.747	−0.036	0.027	0.041	0.968
MHNC	0.042	0.312	0.134	0.893	0.010	0.516	0.430	1.200	0.231	0.125	0.271	0.665	0.408	0.683	0.071	0.244	1.068	0.286
MHP	−0.367	0.443	−0.829	0.408	−0.044	−0.102	0.464	−0.221	0.825	−0.011	−0.919	0.888	−1.035	0.301	−0.122	0.816	1.686	0.092
MIB	−0.051	0.107	−0.477	0.633	−0.039	−0.127	0.104	−1.219	0.223	−0.141	0.405	0.259	1.560	0.119	0.187	−0.532	−6.049	<0.001**
MNP	−0.227	0.335	−0.677	0.498	−0.032	0.045	0.466	0.097	0.923	0.008	−0.205	0.510	−0.401	0.689	−0.026	0.250	0.648	0.517
MOH	−0.809	0.449	−1.803	0.072	−0.273	−0.365	0.562	−0.649	0.516	−0.114	−0.915	0.858	−1.066	0.287	−0.338	0.550	3.196	0.001**
MZP	0.005	0.022	0.210	0.834	0.007	−0.011	0.024	−0.468	0.640	−0.026	−0.005	0.059	−0.090	0.928	−0.004	−0.006	−0.117	0.907

^&^ PFDE, Perfluorodecanoic acid (ng/mL); PFHS, Perfluorohexane sulfonic acid (ng/mL); MPAH, 2-(N-methyl-PFOSA)acetic acid (ng/mL); PFNA, Perfluorononanoic acid (ng/mL); PFUA, Perfluoroundecanoic acid (ng/mL); NFOA, N-perfluorooctanoic acid (ng/mL); BFOA, Br. perfluorooctanoic acid iso (ng/mL); NFOS, N-perfluorooctane sulfonic acid (ng/mL); OP1, Dimethylphosphate (ng/mL); OP2, Diethylphosphate (ng/mL); OP3, Dimethylthiophosphate (ng/mL); OP4, Diethylthiophosphate (ng/mL); OP5, Dimethyldithiophosphate (ng/mL); OP6, Diethyldithiophosphate (ng/mL); CNP, Mono(carboxynonyl) Phthalate (ng/mL); COP, Mono(carboxyoctyl) Phthalate (ng/mL); ECP, MECP phthalate (ng/mL); MBP, Mono-n-butyl phthalate (ng/mL); MC1, Mono-(3-carboxypropyl) phthalate (ng/mL); MCOH, MCOCH phthalate (ng/mL); MEP, Mono-ethyl phthalate (ng/mL); MHBP, Mono-3-hydroxy-n-butyl phthalate (ng/mL); MHNC, MHNCH (ng/mL); MHP, Mono-(2-ethyl)-hexyl phthalate (ng/mL); MIB, Mono-isobutyl phthalate (ng/mL); MNP, Mono-isononyl phthalate (ng/mL); MOH, MEOH phthalate (ng/mL); MZP, Mono-benzyl phthalate (ng/mL). B represents the regression coefficient, where B₁ is the regression coefficient for No-CKD and B₂ is the regression coefficient for CKD. β denotes the standardized regression coefficient. SE (Standard Error) measures the variability of the regression coefficient. The t-statistic is used to test the significance of the regression coefficient, while the p-value determines whether the predictor is statistically significant, with a p-value less than 0.05 indicating significance.

## Discussion

This study delved into the complex relationship between three types of synthetic EDs and renal function. It showed how the association between synthetic EDs and renal function varies with different levels of kidney impairment. This helps us better understand kidney toxicity caused by synthetic EDs. The study found that some chemicals were linked to kidney function damage. These include N-perfluorooctanoic acid and N-perfluorooctane sulfonic acid (from perfluoro/polyfluoroalkyl compounds), Diethylthiophosphate (from phosphorus-containing pesticides), and MECP phthalate, Mono-isobutyl phthalate, Mono-isononyl phthalate, and MEOH phthalate (from plasticizers). The damage levels varied. Further analysis revealed significant differences in the damaging effects between non-CKD and CKD patients. N-perfluorooctanoic acid was significantly associated with renal function impairment in CKD patients. MECP phthalate showed a stronger association with renal dysfunction in non-CKD individuals. This study found that certain perfluoro/polyfluoroalkyl compounds and various plasticizers were associated with renal function impairment. This finding is consistent with previous epidemiological and animal studies, which have observed harmful effects of perfluoro/polyfluoroalkyl compounds on renal function (13–16). These compounds may also be indirectly associated with renal function through a series of complex physiological mechanisms (25–28).

Epidemiological studies have shown that once absorbed into the human body, these compounds primarily accumulate in the serum, liver, and kidney. Previous studies have found that perfluoroalkyl compounds are more likely to accumulate in the kidney than in other tissues (29). The link between perfluoro/polyfluoroalkyl substances and kidney function damage may be because they are very stable chemically. This stability makes them build up in kidney tissues. This accumulation can interfere with various critical signaling pathways and metabolic processes, including fatty acid-binding proteins, peroxisome proliferator-activated receptors and estrogen receptors. These disruptions can cause abnormal fat metabolism, more oxidative stress, and worse inflammation in kidney cells. This can damage kidney tissue structure and affect how the kidneys filter blood and reabsorb substances, leading to renal function decline or failure (30–34). The way plasticizers may be linked to kidney function damage could be by activating biochemical processes. These include problems with mitochondria, DNA damage, abnormal cell growth, and abnormal protein structure. This can damage kidney structure and function, leading to kidney tissue damage and reduced function (35).

Furthermore, our study identified an association between specific organophosphorus pesticides and renal function impairment. This finding aligns with existing evidence demonstrating that exposure to organophosphorus compounds may induce both acute and chronic nephrotoxicity, potentially progressing to renal dysfunction or even end-stage renal disease (36, 37). The possible reasons for this include the ability of phosphorus-containing pesticides to interfere with normal serum albumin metabolism, induce hypoxia, trigger oxidative stress, accumulate lipid peroxidation products, disrupt enzyme system functions, and abnormally activate MAPK signaling pathways. They do this through biological pathways like cholinergic reactions, membrane toxicity, and pro-oxidant reactions. This can ultimately cause kidney tube cell death and kidney function damage (38).

This study revealed significant differences in the damaging effects of various types of synthetic EDs on renal function. Specifically, as the degree of kidney impairment increased, the damaging effects of N-perfluorooctanoic acid among perfluoro/polyfluoroalkyl compounds and Diethylthiophosphate among phosphorus-containing pesticides on renal function exhibited a gradually increasing trend. In contrast, MECP phthalate among plasticizers was primarily associated with adverse outcomes in individuals with less severe renal function impairment. N-perfluorooctanoic acid was especially harmful to the renal function of patients with CKD. While MECP phthalate was more likely to impair the renal function of individuals without CKD (non-CKD).

This difference in effect may be because N-perfluorooctanoic acid has a similar structure to fatty acids. It is also very stable and tends to build up in organs with a lot of fat, like the kidney. This can interfere with important enzymes or receptors in fat metabolism pathways. N-perfluorooctanoic acid affects energy metabolism and the stability of cell membranes in kidney cells. This leads to a lack of energy for the cells and damage to the cell membrane structure. Ultimately, it harms normal kidney function. When kidney cell damage is mild, N-perfluorooctanoic acid builds up less and its effect is weaker. The body’s ability to repair itself is strong. But when kidney cell damage gets worse, N-perfluorooctanoic acid builds up more and the body’s ability to regenerate declines. This makes the harmful effects worse (39). In contrast, MECP phthalate has a relatively stable structure. It mainly affects the normal functions of kidney cells by influencing cell signaling pathways or gene expression. But it builds up less in the kidneys. This leads to a different pattern of kidney toxicity compared to N-perfluorooctanoic acid.

This study has several limitations in exploring the potential association between synthetic EDs and renal function. Firstly, the assessment of synthetic EDs exposure primarily relies on retrospective data or surrogate biomarkers (such as urine markers). Due to the lack of comprehensive exposure history and time-related data, these methods may not accurately capture an individual’s long-term exposure levels. This makes it hard to precisely quantify the dose–response relationship between synthetic EDs exposure and renal function. Secondly, since this is a cross-sectional study, it cannot prove a definite cause-and-effect relationship between synthetic EDs exposure and the observed kidney function damage in CKD and its related conditions. It also cannot completely rule out the possibility of reverse causality or other influencing factors. To elucidate this potential causal relationship, future studies should employ longitudinal cohort studies or randomized controlled trials. Thirdly, the study population may not fully represent the CKD patient group, particularly in terms of demographic characteristics, socioeconomic status, and lifestyle. To address this, future studies should expand the sample size, include more diverse populations, and adequately adjust for potential confounding factors. Finally, this study did not delve into the potential synergistic or antagonistic effects of synthetic EDs on renal function. Future research should fully consider how synthetic EDs interact with each other and how they together affect kidney function outcomes. It should also explore the biological mechanisms behind this.

## Conclusion

This study explored the association between synthetic EDs, particularly perfluoro/polyfluoroalkyl compounds (such as N-perfluorooctanoic acid and N-perfluorooctane sulfonic acid), phosphorus-containing pesticides (such as Diethylthiophosphate), and various plasticizers (including MECP phthalate, Mono-isobutyl phthalate, Mono-isononyl phthalate, and MEOH phthalate), with individual renal function. The results indicate that these synthetic EDs have varying degrees of association with renal function, with distinct differences in their effects. Our analysis revealed that distinct patterns of N-perfluorooctanoic exposure were significantly associated with renal impairment in CKD patients. In contrast, MECP showed associations with renal dysfunction in non-CKD individuals. Therefore, for CKD patients, to prevent further kidney damage from harmful substances like N-perfluorooctanoic acid, we should take comprehensive measures. This includes better medical monitoring of CKD patients, assessing their risk of exposure to products with N-perfluorooctanoic acid, and promoting health education to raise their awareness of harmful substances. This will help them actively avoid or reduce exposure to these substances and protect their kidney health.

This study systematically examined the specific link between various synthetic EDs and individual renal function. These synthetic EDs include perfluoro/polyfluoroalkyl compounds (like N-perfluorooctanoic acid and N-perfluorooctane sulfonic acid), phosphorus-containing pesticides (like Diethylthiophosphate), and various plasticizers (like MECP phthalate, Mono-isobutyl phthalate, Mono-isononyl phthalate, and MEOH phthalate). Our findings demonstrate significant but heterogeneous associations between these synthetic EDs and renal function parameters. Specifically, N-perfluorooctanoic acid was primarily associated with renal function impairment in CKD patients, while MECP phthalate was primarily associated with renal function impairment in non-CKD individuals. In light of this, for CKD patient groups, to alleviate the further harm associated with N-perfluorooctanoic acid to renal function, a comprehensive assessment of CKD patients should be strengthened, meticulously analyzing their risk of exposure to products containing N-perfluorooctanoic acid is needed. Through systematic health education, we should increase the self-protection awareness of CKD patients. This will help them actively avoid or reduce exposure to these substances and effectively protect their kidney health.

In summary, to reduce the potential harm of synthetic endocrine disruptors like MECP phthalate to kidney health, we should widely spread knowledge about the dangers of harmful substances like MECP phthalate through media and the internet. This will raise public awareness of self-protection. Simultaneously, relevant authorities should strengthen market supervision, particularly over daily plastic products that are likely to contain MECP phthalate, ensuring that products on the market comply with safety standards to safeguard consumer health. Additionally, promoting an eco-friendly and safe lifestyle is important. We should encourage the public to use fewer disposable plastic products that may contain harmful plasticizers and choose environmentally friendly alternatives. This will reduce exposure risks to hazardous substances like MECP phthalate at the source and collectively protect kidney health.

## Data Availability

The datasets presented in this study can be found in online repositories. The names of the repository/repositories and accession number(s) can be found at: https://www.cdc.gov/nchs/nhanes/.
